# Functional correlation of bacterial LuxS with their quaternary associations: interface analysis of the structure networks

**DOI:** 10.1186/1472-6807-9-8

**Published:** 2009-02-25

**Authors:** Moitrayee Bhattacharyya, Saraswathi Vishveshwara

**Affiliations:** 1 Molecular Biophysics Unit, Indian Institute of Science, Bangalore – 560012, India

## Abstract

**Background:**

The genome of a wide variety of prokaryotes contains the *luxS *gene homologue, which encodes for the protein S-ribosylhomocysteinelyase (LuxS). This protein is responsible for the production of the quorum sensing molecule, AI-2 and has been implicated in a variety of functions such as flagellar motility, metabolic regulation, toxin production and even in pathogenicity. A high structural similarity is present in the LuxS structures determined from a few species. In this study, we have modelled the structures from several other species and have investigated their dimer interfaces. We have attempted to correlate the interface features of LuxS with the phenotypic nature of the organisms.

**Results:**

The protein structure networks (PSN) are constructed and graph theoretical analysis is performed on the structures obtained from X-ray crystallography and on the modelled ones. The interfaces, which are known to contain the active site, are characterized from the PSNs of these homodimeric proteins. The key features presented by the protein interfaces are investigated for the classification of the proteins in relation to their function. From our analysis, structural interface motifs are identified for each class in our dataset, which showed distinctly different pattern at the interface of LuxS for the probiotics and some extremophiles. Our analysis also reveals potential sites of mutation and geometric patterns at the interface that was not evident from conventional sequence alignment studies.

**Conclusion:**

The structure network approach employed in this study for the analysis of dimeric interfaces in LuxS has brought out certain structural details at the side-chain interaction level, which were elusive from the conventional structure comparison methods. The results from this study provide a better understanding of the relation between the *luxS *gene and its functional role in the prokaryotes. This study also makes it possible to explore the potential direction towards the design of inhibitors of LuxS and thus towards a wide range of antimicrobials.

## Background

**Figure 1:**
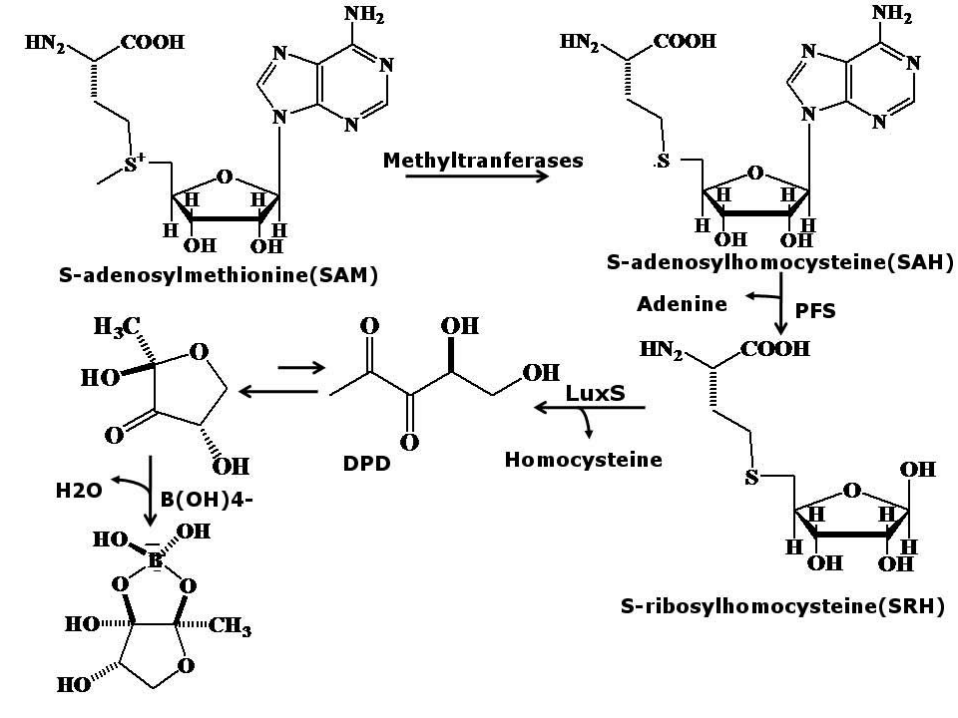
**Biosynthetic path of AI-2 utilizing LuxS to detoxify SRH**.

Quorum sensing is a widespread mechanism of intercellular communication among bacteria controlling its gene expression as a function of cell density. Two major quorum sensing pathways, with characteristic signalling molecules, AI-1 and AI-2, have been identified[1]. LuxS is one of the principal components in the biosynthetic pathway of AI-2, the universal signal for bacterial inter-species communication. In some organisms, quorum sensing by LuxS has been shown to have a profound effect on pathogenicity by affecting toxin production or flagellar morphogenesis and hence motility and colonization. However, in some other species it has no direct pathogenic role and known to affect the metabolism. LuxS is a small metalloenzyme with Zn(II) ion at its active site, which was later predicted to be Fe(II) by Zhu *et al*[2]. Sequence alignment studies have indicated the presence of an invariant HXXEH motif[3] and the metal ion is tetra-coordinated by the side chains of the two histidines of this motif and cysteine, the fourth coordination site being occupied by a water molecule[4,5]. LuxS was shown to exist as a homodimer in its biologically active form by dynamic light scattering studies[6], the two identical active sites being formed at the dimer interface[5]. This protein catalyzes the non-redox cleavage of a stable thioether bond in the SAM (S-adenosylmethionine) cycle to produce AI-2 as shown schematically in Figure 1. Such enzymatic reactions are rare in nature and these enzymes require redox-active cofactors. However, LuxS is unique as it does not require any redox-active cofactor to catalyze this chemically difficult reaction[7].

Protein association plays a dominant functional role such as specific recognition, signal transduction, regulation of gene expression and studies have shown that the symmetry in an oligomer plays an important role as it defines the overall architecture of the protein, and thus its function[8]. LuxS is a biologically active homodimer and extensive research has been carried out to explain the principles underlying protein association, with an aim to characterize the interface[9]. Characterization of the protein-ligand interface using network approach has given important insights into the effects of ligand binding and chain connectivity on network communication in the protein dihydrofolate reductase (DHFR)[10]. The dimerization domain was shown to be very important in relation to the structure and function of LuxS as is evident from the occurrence of the active site at the dimer interface. Yet another evidence for the importance of the homodimer is apparent from the studies of electrostatic potential and conserved residues on the monomer surface[5]. The crystal structures of LuxS from only four organisms are available in the PDB in their dimeric states, and it has been proposed to contribute a unique fold to the alpha-beta family of proteins[5,11]. Interestingly the protein structures superpose very well at the backbone level (Additional file 1: Table S2) and this information does not provide any clue to the diversity in the function of LuxS. In the present study, we have highlighted the intricate details of side chain interactions at the dimeric interface, in order to distinguish LuxS structures from different sources.

Graph theoretical approaches have proved to be useful in capturing the side-chain interactions in a collective way through the representation of protein structures in the form of protein structure networks (PSN)[12]. For example, the analysis of the interface clusters in PSN has given insights regarding the sequence signature motifs responsible for the different types of quaternary association in legume lectins[13]. Also, structural domains and domain interface residues in multi-domain proteins were successfully identified by graph spectral analysis[14]. Protein network analysis has also provided insight into the domain-domain interactions in proteins in the context of allostery[15]. The importance of minor side-chain variations in the light of 'allostery without a backbone conformational change' has also been established[16]. Recently, crucial residues involved in the oligomerization of *M. smegmatis *Dps were predicted from graph based method and has been experimentally proven to be crucial for the functional oligomeric state of the protein[17]. The aim of the present study is to exploit the advantages of PSN graphs for the characterization of the interface in case of LuxS from bacteria of various species. Our study has revealed the potential of PSN graphs as an analytical tool in judging differences in side-chain interactions; the backbone level having almost no differences at all. The interfaces are characterized in several selected structures (X-ray structures as well as the modelled ones) and the results are extended to LuxS family of proteins in general. An indirect correlation between the dimeric interface organization and the biological function such as pathogenesis is brought out from these studies. Additionally, potential sites of mutation at the interfaces are also identified. Some of them are shown by previous studies to be structurally and functionally important residues in LuxS.

## Results

Crystal structures of LuxS from four organisms (*H. pylori, H. influenza, D. radiodurans and B. subtilis*) are available in PDB[5,11]. Structures of LuxS dimers from 19 other organisms are modelled as described in the Methods section and our dataset is divided into six distinct classes (Table 1) on the basis of the biological activity of the organism and/or the proposed effect of LuxS on pathogenesis. Interface cluster analysis is performed on the PSNs of LuxS from these 23 structures and the results are presented below.

**Table 1:** DATASET

CLASS	NAME OF ORGANISM	SwissProt ID	PDB_id	ABBREVIATIONS USED
**I**	*H. influenzae*	P44007	1j6w	1j6w
	*S. epidermis*	Q8CNI0	-	m1ste
	*S. mutans*	Q8DVK8	-	m1stm

**II**	*H. pylori*	Q9ZMW8	1j6x	1j6x
	*E. coli*	Q8X902	-	m1eco
	*C. jejuni*	Q9PN97	-	m1cam
	*S. aureus*	Q6GEU1	-	m1sta

**III**	*B. subtilis*	O34667	1j98	1j98
	*B. anthracis*	Q81KF3	-	m1bac
	*B. clausii*	Q5WDW1	-	m2bac
	*B. cereus*	Q816N5	-	m3bac

**IV**	*D. radiodurans*	Q9RRU8	1inn	1inn
	*D. geothermalis*	Q1IW42	-	m1geo
	*P. ingrahamii*	A1SZZ2	-	m1psy
	*T. thermophilus*	Q72IE6	-	m1the

**V**	*V. cholerae*	Q9KUG4	-	m1vib
	*C. perfringens*	Q0SWJ6	-	m1clo
	*S. pyogenes*	P0C0C7	-	m1pyo
	*S. flexneri*	Q83JZ4	-	m1shi

**VI**	*L. johnsoni*	Q74HV0	-	m1lac
	*L. reuteri*	Q5QHW1	-	m2lac
	*L. acidophilus*	Q5FK48	-	m3lac
	*B. longum*	Q8G568	-	m1bfi

Classification of LuxS from 23 organisms on the basis of their effect on pathogenesis and/or biological manifestations. I – LuxS mutation affects biofilm formation; II – LuxS mutation affects motility/flagella formation/metabolism; III – LuxS from organisms belonging to the *Bacillus *sp.; IV – LuxS from extremophiles; V – LuxS mutation affects toxin production; VI – LuxS from probiotics. The abbreviations for the modelled structures are preceded by an 'm' to indicate that these structures are modelled using MODELLER.

### Interface cluster analysis of LuxS

**Figure 2:**
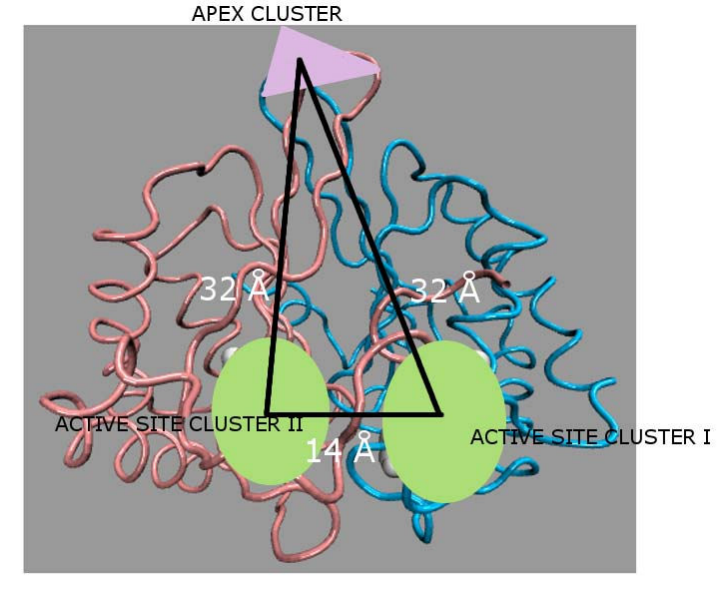
**Schematic representation of the triangular (isosceles) orientation of the active site (I/II) and apex clusters**. The active site clusters I and II (shown by light green oval shaped region) and the mini-triad (shown as a mauve triangle) at the apex of the dimer interface for the protein LuxS (Average edge lengths are given); the protein backbone shown in tube representation with the two chains coloured differently.

**Figure 3:**
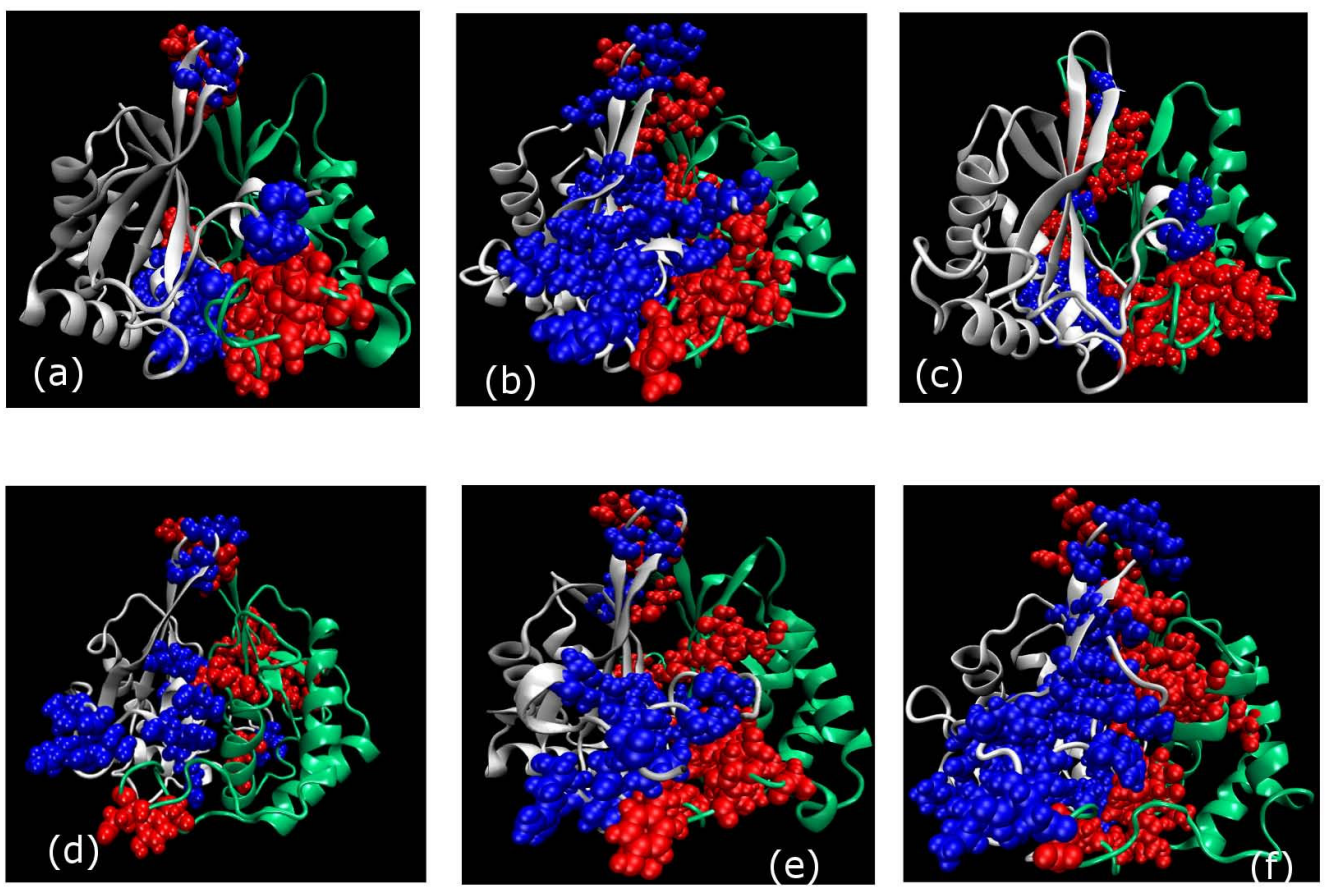
**All Interface amino acid clusters in one representative member from each class (I–VI)**. (a) CLASS I (*H. influenzae*), (b) CLASS II (*H. pylori*), (c) CLASS III (*B. subtilis*), (d) CLASS IV (*D. radiodurans*), (e) CLASS V (*V. cholerae*), and (f) CLASS VI (*L. johnsonii*), at I_min _= 6%. The two subunits of the LuxS dimer are represented in different colours (chain A in white and chain B in green) by new cartoon representation. The cluster forming residues are shown in van der Waal's representation (Blue spheres and red spheres represent the residues from chain A and chain B respectively).

The protein dimer is considered as a connected network of nodes and edges as described in the Methods section. The side chain interactions at the dimer interface are captured at different threshold values of interaction strength (I_min_). The number of interface clusters varies from 4 to 12 (details of each class at I_min _= 6% is given in the Additional file 1: Table S1). However, three of them are consistently present in most of the structures in geometrically equivalent positions, occupying the three vertices of an isosceles triangle as shown schematically in Figure 2. Two of the clusters (active site clusters) host the active site residues and the third one (apex cluster) is at the apex of the dimer with the exposed loop residues arranged in a mini-triad form. Cartoon representations of all the interface clusters (ranging from 4 to 12) at I_min _= 6% are shown for one representative member for class (**I–VI**) in Figure 3(a–f) and the residues present in these interface clusters are summarized in Additional file 1: Table S3. It is interesting to see a different pattern of interface clustering in terms of the size of the cluster and its distribution across the interface in each class (**I–VI**) in spite of the fact that their backbone RMSDs (Table S2) are considerably small. For instance, it is noted that the maximum similarity at the backbone level among the four PDB structures of LuxS is observed between *H. pylori *and *B. subtilis *(rmsd = 0.778). However, their interface cluster representations show prominent dissimilarities in size and distribution as shown in Figure 3(b, c); the *B. subtilis *interface cluster being of approximately half the size of that of *H. pylori*. Although, the total number and size of interface clusters also vary within a class, but the overall topological distribution of the amino acid residues at the interface within a class is uniform as depicted pictorially for class **II **and **III **in Additional file 2: Figure S1(a-b) respectively. Thus, we find that our interface clusters exhibit certain characteristic properties that can be related to the biological activities, which will also be elaborated more in other sections.

### Distribution of Active Site residues in the Interface Clusters

**Figure 4:**
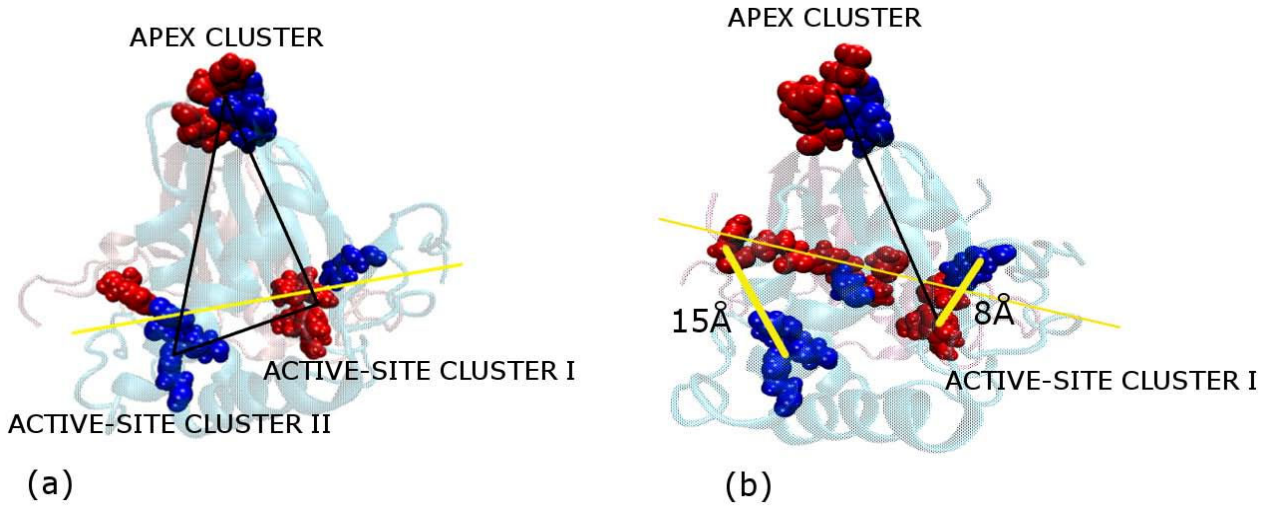
**Triangular pattern at the dimer interface**. The pattern comprises of the two active sites clusters I and II and the apex cluster at the top vertex of LuxS. (a) Isosceles triangular pattern of the amino acid residues in the interface clusters (cluster I, II and apex cluster) well-organized at the dimer interface for m1eco. The edges of the triangle are calculated to be 31.66, 31.42 and 13.09 Å for **active-site cluster I, apex cluster, and active site cluster II **moving clockwise, (b) Isosceles triangular pattern of the amino acid residues in the interface cluster is distorted at the dimer interface for the probiotics and some extremophiles, as shown here for the probiotic m1lac. The edge between **apex cluster and active site cluster II **is 31.33 Å but **active-site cluster I **is missing from the interface cluster as the three His (chain A) have moved away from Phe (chain B) by 15 Å as compared to 8 Å distance between Phe (chain B) and the three His (chain A). The backbone is represented by transparent new cartoon and the amino acid residues at the vertices of the triangle as van der Waal's spheres; each monomer and its amino acid residues coloured differently. Residues from chain A are coloured Blue and those from B are coloured Red. An imaginary line is drawn across the interface in yellow and the distances between His 58 (CB) and Phe 7 (CB) are approximately given for m1lac.

LuxS has been reported to contain two identical active sites at the dimer interface including residues from both the subunits. The properties of LuxS in terms of active site, invariant sequence motif (His-Xaa-Xaa-Glu-His) and the metal ion coordination has been discussed in the Background section. Our investigation focuses on the distribution of the active site residues in the interface clusters. The results indicate that the active-site interface clusters reproduced the similar symmetric distribution of the active sites at the dimer interface in most of the classes [Figure 4(a)]. However, the probiotics (class **VI**) and some of the extremophiles (class **IV**) exhibit different type of association where specifically only one of the active site cluster is part of the interface cluster whereas the second active site cluster is made by the residues of only a single subunit. This is shown pictorially in Figure 4(b).

A general geometric pattern of two active site interface clusters and the mini-triad apex cluster is noted at the interface for classes (**I–II and V**). The active site clusters are made up of residues (three His and one Glu) from one subunit and a Phe residue from the other subunit. Thus, the active site of subunit A is supported by the Phe residue of subunit B and *vice versa*. Mini-triad in the apex cluster mentioned here is found to be unanimously present in our interface clusters for all the classes (**I–II, IV-VI**) at high side chain interaction strengths (I_min _= 6%) except for the LuxS from *Bacillus sp. *(class **III**) in which such mini-triad is absent from the interface clusters.

On close inspection of the mutual orientation of the three invariant interface clusters, it is noted that they arrange themselves in the form of an isosceles triangle with comparable distances as shown in Figure 4(a). However, in probiotics (class **VI**) and some extremophiles (class **IV**) it is noted that this triangular pattern on the interface is distorted as shown in Figure 4(b), either by the absence of the three His or the Phe or both from the interface clusters.

The results indicate that the isosceles triangular pattern at the interface is violated [as shown in Figure 4(a–b)] in three cases: class **III **in which the mini-triad is absent from the interface cluster and class **VI **and some members of class **IV **in which the interface clusters contained the active site only from one subunit (Table 2, Additional file 1: Table S3).

**Table 2:** Active site residue participation at the dimeric interface clusters of LuxS

	**ClassI**			**ClassII**			**ClassIII**			**ClassIV**			**ClassV**			**ClassVI**	
	**6%**	**8%**		**6%**	**8%**		**6%**	**8%**		**6%**	**8%**		**6%**	**8%**		**6%**	**8%**

1j6w	**	**	1j6x	**	**	1j98	**	+	1inn	+	+	m1vib	**	-	m1lac	+	-

m1ste	**	**	m1eco	**	**	m1bac	**	**	m1geo	**	-	m1clo	**	-	m2lac	+	+

m1stm	**	**	m1cam	**	**	m2bac	**	+	m1psy	**	**	m1pyo	+	-	m3lac	+	+

			m1sta	**	**	m3bac	+	+	m1the	+	+	m1shi	**	**	m1bfi	+	+

Active site residue participation at the dimeric interface clusters of LuxS as evaluated at two interaction strength of I_min _= 6% and 8% from classes I to VI. + refers to the presence of either of the active site cluster I or II in the interface clusters, – means active site clusters are completely absent from interface clusters, ** means both active site cluster I and II are present in the interface clusters.

### Interface signature motifs

The interface cluster analysis enables the identification of sequentially apart, but structurally proximal residues. As mentioned above, the three clusters (two active site clusters and one apex cluster) are common to most of the structures. In addition, interface clusters at other locations are also present in most cases. Here we have analysed the occurrence of residues at the interface coming from structurally similar positions in all the interface clusters. The residues are generally conserved completely or partially within a given class emphasizing its evolutionary importance for organisms within a particular class. However, the His and the Phe residues in the active site clusters are completely conserved across all the families. Such an analysis of position specific residues in the interface clusters brings out the class specific signature motifs of the interfaces. The results are summarized in Table 3 and they are pictorially represented in Figure 5(a–b). From the table it is evident that most of the residues appearing in the signature motif are conserved either completely or partially within the class. However the residues in the mini-triad which was discussed previously are generally not conserved. Interestingly, it is observed that the mini-triad motif is absent from the class **III **signature motif in contrast to the other classes. The signature motif is plotted and marked on the superposed backbones of the all members of a particular class for class **V **and **VI **in Figure 5(a–b) respectively.

**Table 3:** Description of the interface signature motifs.

CLASS*	FRACTION OF CONSERVED/PARTIALLY/NON CONSERVED RESIDUES AT THE INTERFACE FORMING THE MOTIF**	^#^**SEQUENCE NUMBER AND RESIDUE NAMES OF THE SIGNATURE MOTIF**
**II**	27/242 (11.16%)	**5 *V/L(A/B) *8F(A/B) **28G/T(A/B)**30 *N/H/K(A/B) *32D(A/B) **116A/E(A/B)**55H(A/B) 59H(A/B) 128H(A/B)**

**III**	24/206 (11.65%)	**4V(A/B) 7F(A/B) 57E(A) 83G(A) 120A(A/B) 54H(A/B) 58(A/B) 132H(A/B)**

**IV**	30/183 (16.39%)	**10F(A) **30T(A/B) 32K/R/S(A) 34D(A/B)**76D(A/B) 87Y(A/B) *117I(B) *57H(B) 61H(B) 131H(B)**

**V**	33/218 (15.14%)	**7F(A) 10 D(B) **27 T/G(A/B)**29 *K/H(A/B) *31D(A/B) 55T(B) 57E(B) 84 *R/K(A/B) *54H(A/B) 58H(A/B) 134H(A/B)**

**VI**	45/214 (21.02%)	28Q/P(A)**29 *K/H(A/B) *31D(A/B) 33I(A) 57E(B) 72I(B) 77F(A) 80R(B) 84H(A) 88W(A/B) 126N(A) 129D(A) 54H(B) 58H(B) 130H(B)**

The numbering of residues is done for one representative member of each class (1j6x-class II, 1j98-class III, 1inn-class IV, m1vib-class V, and m1lac-class VI). Complete conservation of the residues is indicated in **bold **and the ***bold italics ***indicate semi-conservative mutation within the class; the normal font indicates no conservation within the class. Fraction of conserved/partially/non conserved residues at the interface that forms the interface signature motif is also given. The conservation information is based on the proteins taken in this study and within a class (approximately the residue conservation was verified to be similar in a larger dataset of available sequences in classes such as III and VI).

* Any definite motif was absent in class I

# (A/B) refers to the residues from both the chains. (A) or (B) refers to the residues from a single chain.** (Number of conserved/partially/non conserved residues forming the motif)/(total number of interface residues).

**Figure 5:**
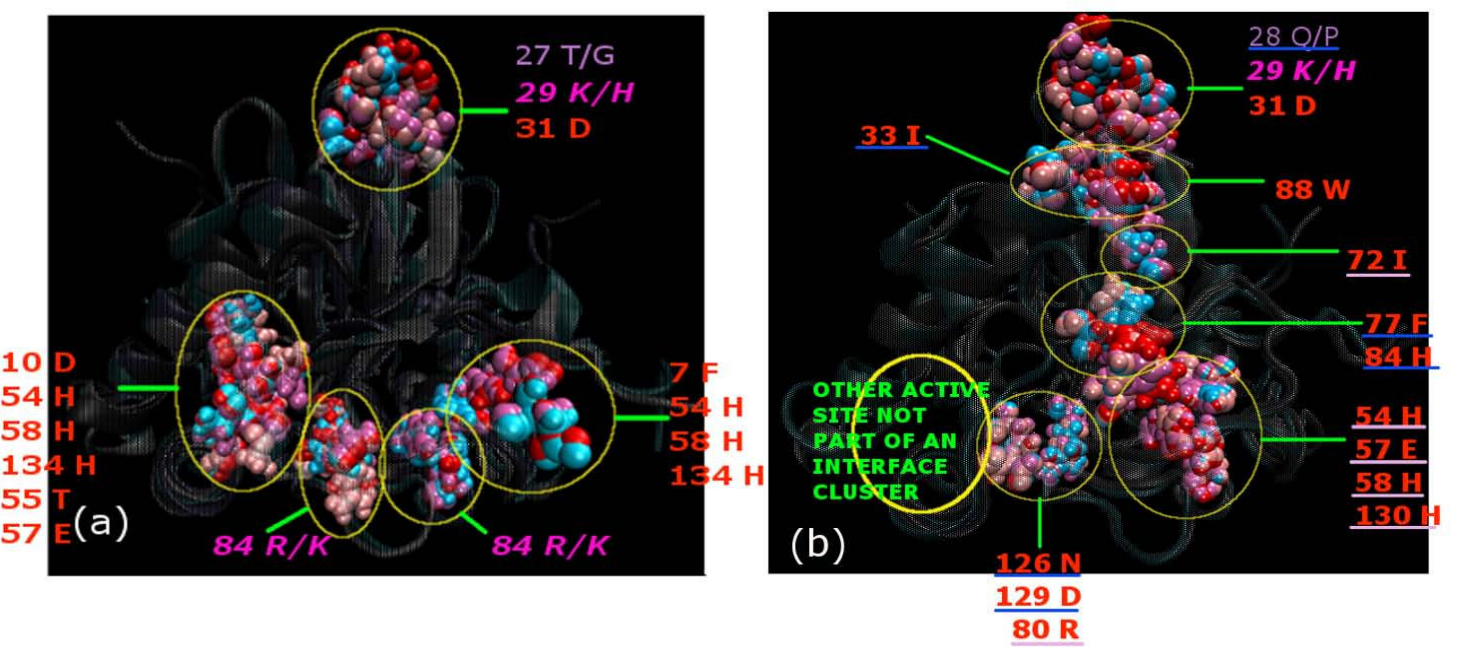
**Interface signature motif**. The signature motif for (a) CLASS V (m1vib, m1clo, m1pyo, and m1shi), and (b) CLASS VI (m1lac, m2lac, m3lac, and m1bfi); based on superposition of all the LuxS structures belonging to a particular class and prediction of structurally superposing residues (by manual inspection) as depicted pictorially to form a motif at I_min _= 6%. The superposed backbone structures are represented as transparent new cartoon and the interface cluster residues forming the motif are shown as van der Waal's spheres, specific colour represents residues from a specific protein within the class. The different regions are marked and labelled with the corresponding residues in different colours; red/pink indicates completely/partially conserved residues respectively within the class, whereas violet indicates no conservation within the class. The completely/partially conserved residues are in **bold/*bold italics ***respectively. The residues coming from only one subunit are underlined in blue (chain A) and mauve (chain B).

### Apex cluster at the dimer interface (Mini-triad motif)

**Figure 6:**
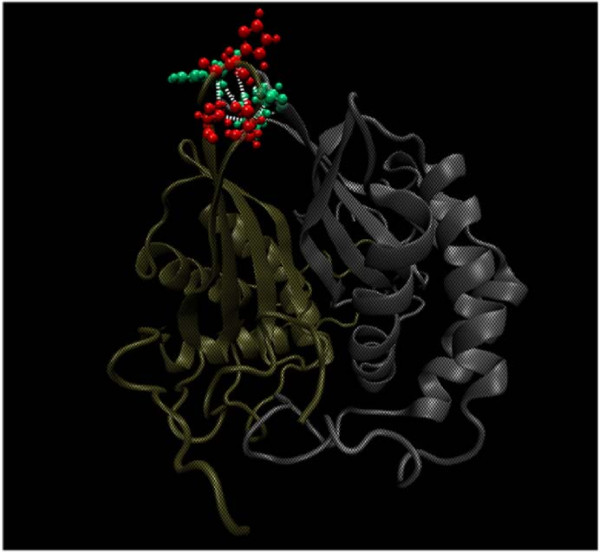
**Apex cluster at the dimer interface**. The triads formed from sequentially apart residues (x (A/B), x+2 (A/B), x+4 (B/A), where x is an arbitrary sequence number) at the top vertex found for all LuxS proteins under study (except for *Bacillus sp. *LuxS). The triad for 1j6x (class II) is depicted here; the backbone is represented as new cartoon and each monomer and the residues coming from it are coloured differently.

**Figure 7:**
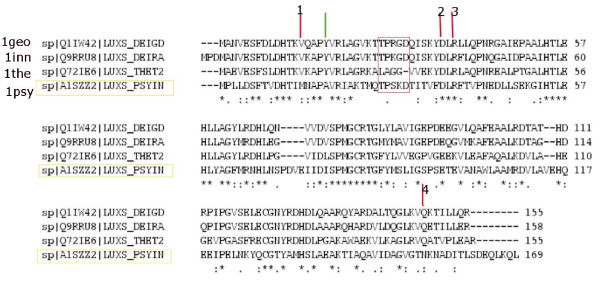
**Representation of Multiple sequence alignment (using ClustalW) within class IV**. The "persistent hub" and the four connecting residues are highlighted in the figure with coloured lines; the green arrow indicates the "persistent hub" and the red arrows indicate the connecting residues and are numbered arbitrarily from 1 to 4. Residue 2 and 3 are completely conserved within the class whereas residue 4 is partially conserved. However the "persistent hub" and the residue 1 are completely conserved within class IV excluding m1psy. The region marked in the orange box indicates the residues present in the mini-triad for class IV; the mini-triad sequence being absent from m1the.

A triad cluster (Figure 6) formed by the amino acids belonging to a stretch of five residue sequence, with two of the residues arising from one subunit and the third one coming from the other subunit is identified in LuxS protein dimers. The general sequence representation of the triad is x (A/B), x+2 (A/B), and x+4 (B/A) where x represents an arbitrary residue number; A and B represents the two subunits of the protein. There is substantial variation in the type of the amino acids in these motifs. However, a careful examination has revealed consistency in the amino acid type belonging to a particular class (the details of the participating residues are given in Additional file 1: Table S1). This repeating triad forms a kind of bridge between the two subunits of the dimeric protein since one of the three residues in this pattern always comes from the other chain; and this triad always appears in our interface clusters even at high interaction cut-offs. Such an interface triad is identified in all classes with the exception of the *Bacillus sp. *(class **III**). Also, for m1the in class **IV **the mini-triad motif is absent from the sequence itself as becomes evident from the multiple sequence alignment in Figure 7.

**Figure 8:**
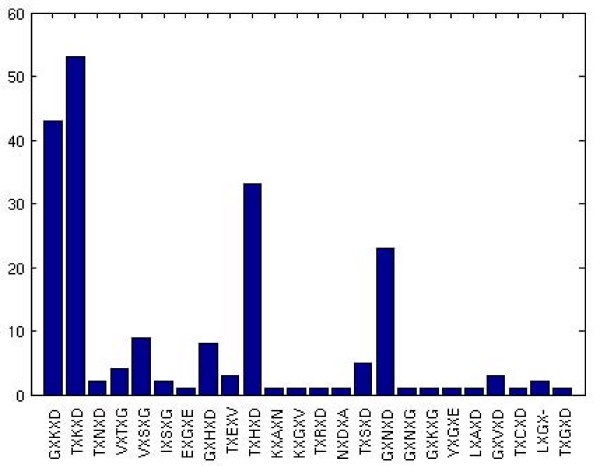
**Frequency of occurrence (as shown in the Y-axis) of the predicted sequentially non-conserved mini-triad motifs (details shown in the X-axis) in a dataset of 202 proteins from the LuxS family. '**X' indicates any amino acid residue.

The triad motif identified at the dimer interface from the structures is searched in the sequence space of 202 LuxS proteins from different organisms. 24 different patterns are found for the triads and we found the frequency of occurrence of each pattern in the dataset and plotted them as shown in Figure 8. These motifs are majorly conserved within a bacterial species as becomes evident from Additional file 1: Table S5 and Additional file 3: Figure S2; for instance, GXKXD is mainly present in the *Streptococcus sp*. and *Lactobacillus sp*. in our dataset. These motifs are present in the interface clusters of most of the classes which we have selected for analysis except for class **III **(*Bacillus sp.*), in which the motif is VX(T/S)XG and it is not a part of the interface cluster. The general chemical nature of the triad motif for our dataset is found to be G/polar(x), Basic/polar(x+2) and Acidic(x+4). This triad motif is violated only in case of two classes: the mini-triad motif is not very uniformly retained in case of the interface clusters of the probiotics with the xth residue (Gly) being usually replaced by a polar (x+1)th residue. Also for the *Bacillus sp*. the acidic(x+4) residue is replaced by Gly and the xth residue is usually Val. Such a difference in sequence motif is indeed correlated with the structure motif. Strikingly, the *Bacillus *family LuxS proteins lack the mini-triad in the apex cluster at the interface.

### Hub Analysis and Extremophiles

**Figure 9:**
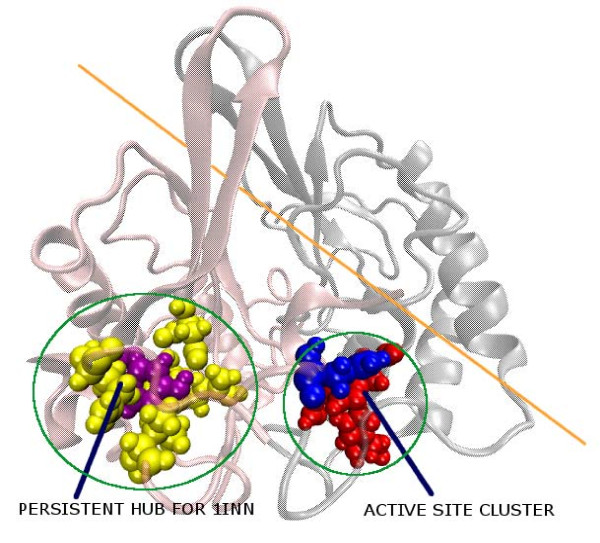
**Representation of the "persistent hub" present at I_min _= 12% for **1inn**(class IV) LuxS**. The active site cluster, the hub and their connecting residues are represented by van der Waal's spheres. The hub is coloured purple and the connecting residues are coloured yellow. Blue spheres depict residues from chain A and red represents those from chain B in the Active site cluster. The protein backbone is shown in new cartoon representation with the two subunit coloured differently. An imaginary line is drawn across the interface in orange.

The extremophiles deserve a special mention in terms of the hub analysis, the details of the method given in the Methods section. It is noted that for the extremophilic LuxS proteins in our dataset (class **IV**) one particular characteristic core (at non-interface region) hub is retained at very high cut-offs (I_min _~ 12%) for all the members of this class as shown in the Additional file 1: Table S4 except m1psy (psychrophile) in which no hubs are detected beyond I_min _= 8%. Hubs at such high I_min _values are not detected for members from any other class in our dataset. This "persistent hub" (16Y for 1inn) and the four other connected residues (12V, 35D, 37R, and 141Q for 1inn) and their analogues in the other members of this class (m1geo and m1the) are thus identified as characteristic of the extremophilic class of LuxS proteins. One reason for the absence of the "persistent hub" from m1psy is that in its sequence Tyr (the "persistent hub") is mutated to Ala. The multiple sequence alignment[18] within class **IV **shows that the hub and its connecting residues are mostly completely/partially conserved as shown in Figure 7. The hub is identified as an important site for mutation and its location (along with its connecting residues) with respect to the active site cluster in 1inn is shown pictorially in Figure 9, which reveals its vicinity to the active site. It should be noted that the sequence of the radiation-resistant bacteria *D. radiodurans *(1inn) and *D. geothermalis *(m1geo) and the hyperthermophile *T. thermophilus *(m1the) are more similar to each other than that of the psychrophile *P. ingrahamii *(m1psy) (as is shown by Figure 7[18]) which is also reflected in the hub and its connecting residues.

### Identification of key residues at the interface by Graph theoretical analysis

**Figure 10:**
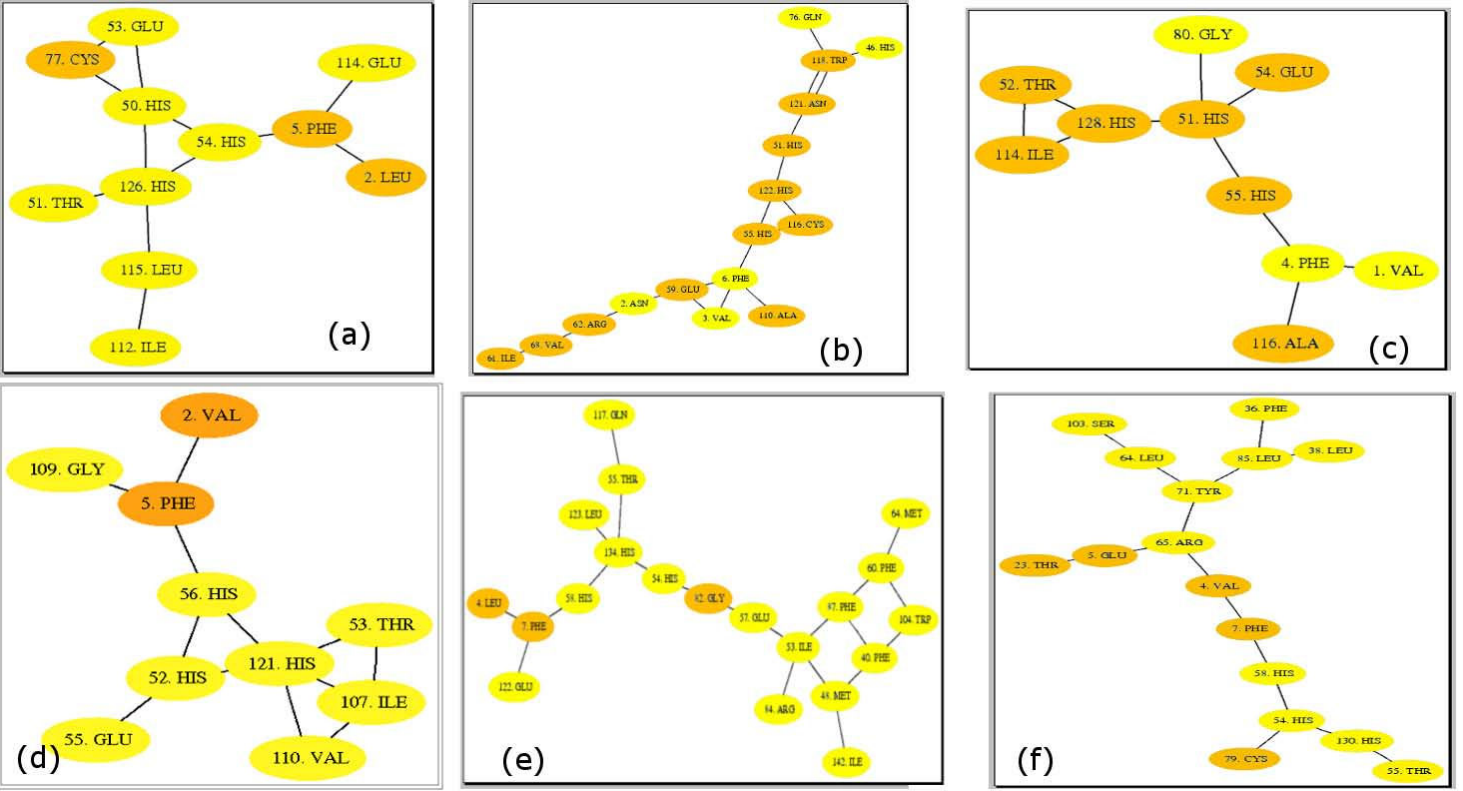
**(a-f): Connectivity representation (drawn using Graphviz**[19])** of the residues in the largest interface cluster as obtained from graph theoretical analysis**. One representative member is chosen from each class: (a) class I (1j6w), (b) class II (1j6x), (c) class III (1j98), (d) class IV (1inn), (e) class V **(m1shi)**, and (f) class VI **(m1lac) **respectively. The residues from chain A are coloured orange and those from chain B are coloured yellow.

Graph theoretical analysis is useful in understanding the role of the residues in holding the integrity of the interface. The nature of connections among amino acid residues in the largest interface cluster hosting one of the active site clusters are depicted in Figure 10(a–f)[19] for a representative member from each class (**I–VI**) respectively. Mostly, the interface cluster containing the second active site [the second active site cluster being absent from the interface clusters of the probiotics and some extremophiles as *D. radiodurans *(1inn)] has lesser amino acid residues than the one described above, thus reflecting some asymmetry in the size of the two active site containing interface clusters. Also, there is prominent asymmetric distribution within the clusters, with the majority of amino acid residues coming from one chain and being supported by only few residues from the other chain in the dimer.

It is evident from the connections derived among the amino acid residues for the two active site containing interface clusters that apart from the immediate active site residues, other residues like Phe and Glu in the vicinity of the active site also play a major structural and functional role. The Glu residue is highly connected in the active site interface clusters and appeared to be at the cluster centre in some cases (which is also quantitatively evaluated from the graph). The participation of Glu in the active site interface cluster also assumes importance by the fact that in the catalytic reaction of LuxS, Glu plays a major role as a general acid/base as has been shown earlier by mutation studies. For class (**I–III **and **V**), the active sites on either subunit contain a closely connected network among all the three His, and Phe and Glu near the active site at I_min _= 6%. So this ensemble of three His, and Phe and Glu near the active site is identified to be necessary for LuxS function in relation to pathogenesis in these classes. However, for the probiotics (class **VI**) and some extremophiles (class **IV**) [e.g. 1inn as shown in Figure 10(d)] only one subunit has the three His and Phe in the interface clusters; the other subunit does not present the active site at all as a part of the interface cluster at I_min _= 6%. We propose that both the active sites and the important residues in its vicinity are required for the proper functioning of the protein in relation to pathogenesis.

### Potential sites of Mutation

From crystallographic studies by Lewis *et al*[5] it was known that Glu57, Arg65 and Asp78 (sequence positions for *B. subtilis *LuxS) were important for substrate binding in LuxS. This is supported by our results of interface cluster analysis. Moreover, Glu57 is sequentially highly conserved, and was proposed to have a role as a general acid/base in the mechanism of LuxS action. It was shown by Zhu *et al*[2] that mutation of Glu57 in *B. subtilis *LuxS to Ala57 and Gln57, the LuxS activity was completely lost, indicating the importance of Glu57. This is also observed from our analysis which shows that for *B. subtilis*, Glu57 is connected to the active site residues [as shown in Figure 10(c)] in one of the interface clusters in 1j98 and its mutation would thus definitely distort the active site geometry.

The interface cluster analysis enables prediction of amino acid residues apart from the active site that are potential sites of mutation; this would warrant experiments to verify them. We propose that for class **V **of LuxS proteins, the residue Arg84 (for m1vib) and its analogues in the other structures play the role of the second general acid/base in the catalytic reaction. Similarly, for class **VI**, this function is attributed to Arg80 (sequence number for m1lac). So it is proposed that their mutation is likely to render the LuxS protein inactive similarly as was shown previously by Zhu *et al. *for *B. subtilis *LuxS. Also, the repeated occurrence of the sequentially conserved Phe8 (sequence number for 1j6x) and its analogues in the interface clusters for all the structures at various interaction strengths asserts its importance. On inspection of its position in the structure, we found it stabilising the active site core through π-stacking interactions. So these are potential sites of mutation and calls for experimental validation. Also the amino acid residues at the mini-triad identified at the apex of the protein structure are potential sites of mutation.

The extremophilic class (class **IV**) is shown to retain one particular characteristic core hub as described previously at very high cut-offs (I_min _~ 12%) and this hub residue (Tyr) is thus proposed to be an important site for mutation experiments as well.

## Discussion

Bacteria have efficiently imbibed mechanisms for communication within themselves and the environment. Quorum sensing is a mode of such crosstalk as a function of cell density and has been divided into two categories (quorum sensing system 1 and 2) with their characteristic signalling molecules, autoinducer 1 and 2 respectively. LuxS is the precursor protein of autoinducer 2. Although the quorum sensing, in general, can directly influence pathogenicity by controlling bacterial gene expression as a function of cell density, it is the unique feature of LuxS induced quorum sensing system to control bacterial metabolism. So LuxS driven quorum sensing has the ability to control not only pathogenicity but also the 'overall well-being' of the organism. The effect of LuxS mutation manifests in different ways in different organisms. As an example, for the organisms like *H. influenza, S. epidermis*, and *H. pylori, E. coli*, it has been shown that mutation of LuxS affect biofilm development and metabolism/motility/flageller morphogenesis[20,21] respectively (affecting metabolism), whereas for organisms like *V. Cholera, C. perfringens*, the effect was shown to be related to toxin production[21-23] (direct influence on pathogenicity).

In the present study we have analyzed the quaternary association features of the available and modelled structures of LuxS from 23 different organisms. Our study indicates the presence of distinct key dimer association features in the LuxS protein from different species related to the biological manifestation of the protein. This fact has enabled us to classify our dataset into six categories and perform a detailed analysis in order to characterize their interface. A simple analysis of the protein structures over our dataset at the backbone level is largely elusive and predicts the structures to be highly similar. However, finer differences exist at the side-chain level, which has become evident through our analysis of the interface clusters. A detailed description of cluster sizes, number of residues in the clusters is provided in the Additional file 1: Table S1. There is a wide variation of the interface cluster size and their orientation, although their backbone rmsds are comparable (Additional file 1: Table S2), which accounts for the range of finer diversities existent within the structure of the same protein from different organisms.

Our analysis has also enabled the prediction of signature motifs of completely/partially conserved sequentially apart but structurally proximal residues at the dimer interface for each class. Since these signature motifs appeared because of their spatial proximity, it would not have been possible to identify these motifs by sequence comparisons alone. Based on similar cluster analysis studies, it was predicted previously that the quaternary association of lectin from *Lotus tetragonolobus *did not correspond to any known type of association pattern[24] and indeed a new type of association was later discovered for this protein by crystallographic studies[25]. This type of analysis would at least enable, in this case, to predict, *a priori*, the probable physiological function of the LuxS protein from a given organism by superposition of interface clusters (described in details in the Methods section) and comparison of the motif obtained with the signature motifs from the different classes. These identified motifs can serve as possible epitopes for LuxS inhibitor design as they are highly vulnerable structurally conserved regions of the protein within a particular class. However, for organisms sharing the same niche, e.g. belonging to the enterobacterial group, like *Helicobacter pylori, Escherischia coli *and *Salmonella typhimurium *(results not shown), it becomes exceedingly difficult to target the LuxS of a particular organism without any effect on the other. On close inspection of the side chain interface clusters of these three organisms, it was found that they are mostly superposing implying a very large similarity in their structures even at the side chain level. So it is not possible in such cases to target one without influencing the other. However, as discussed later in the context of the mini-triad motif, some level of discrimination is possible.

Our study reveals the presence of a triangular pattern at the interface that is constituted by three most important clusters as shown in Figure 2. The probiotics and some extremophiles and class **III **members manifest an exception as this geometric pattern is distorted in them. Probiotics are beneficial bacteria and includes *Lactobacillus*, and *Bifidobacterium *species. They possess numerous potential therapeutic properties including anti-inflammatory and anti-cancer activities and other features of interest[26]. In recent years, studies with *in vitro *cell culture and animal models clearly demonstrated protective effects of probiotics for anti-tumor and anti-cancer effects[27]. It was shown previously that *L. acidophilus *secreted molecule(s) that either inhibited Quorum sensing signal or underwent direct interaction with bacterial transcriptional regulators, controlling the transcription of enterohemorrhagic *E. coli *O157 genes involved in colonization[28]. However, both the genomes have the *luxS *gene homologue. It was also proposed that AI-2 might interfere with the normal functioning of commensal organisms in the gut by competing with their communication and thus, taking colonization advantage[29]. So it is intriguing to note the difference of the geometric pattern of distribution of the active sites on the dimer interface as is captured by our interface cluster analysis. It is striking that the probiotics actually contained the LuxS protein that contributed to virulence and related phenomenon in several bacterial species. Thus, it is proposed that this geometric pattern at the dimer interface might play an important role in the behavioural outcome of LuxS in relation to its function; absence of which distinguishes the probiotics from the rest of the organisms considered in our study in terms of function.

Another important observation from our analysis is the presence of a mini-triad at the apex of the dimer which presented itself in the interface clusters throughout for all the structures in our dataset except for class **III **(LuxS from *Bacillus sp*.) in which it is absent from the interface cluster. However, this set of residues does not show up as conserved in multiple sequence alignment studies of the 23 proteins in our dataset. Its position in the structure at the apex of the dimer and also the repeated occurrence in the protein interface cluster indicate that it might be an important site for mutation; we hypothesize that their mutation might reorient or distort the dimerization interface. Also, we find that there is a marked difference in the chemical nature of the residues constituting the mini-triad motif between *H. pylori *(GXNXD) and that of *S. typhimurium *and *E. coli *(TXHXD), although they share a very high similarity even at the side-chain level. This mini-triad is a highly accessible target for small molecules as they are in the exposed regions of a loop at the apex of the protein dimer. These small molecules may be potential drugs leading to a perturbation at the dimer interface, targeting specifically *E. coli *and *S. typhimurium *LuxS but not *H. pylori *LuxS.

Graph theoretical analysis is helpful to visualize the actual global connectivity in the protein structure network at a given interaction strength. From this analysis, we predict that it is an ensemble of amino acid residues that dictates the function of LuxS. So we predict few potential sites of mutation in the LuxS protein. Some of our predictions of possible sites of mutation have been already experimentally justified; some would warrant experimental verification.

The results of hub analysis reveal a special characteristic feature in the extremophilic class (class **IV**); the recurrent presence of a core hub at a high interaction strength (I_min _= 12%). Furthermore, the asymmetry of the interface discussed before also manifests within the core of the protein as shown by the presence of the "persistent hub" in one chain and not in the other. It was shown previously that hubs in real world networks provide robustness to such networks[30]. Previous studies have also demonstrated that the thermophilic proteins contained more hubs than their mesophilic analogues[31] and these hubs were found to be important for the overall integrity of the structure of such proteins. The "persistent hub" and its connecting residues are thus recognized as potential sites for de-stabilizing mutations in the LuxS proteins from extremophiles (class **IV**).

The LuxS family of proteins (there are 202 proteins with annotated sequences in UNIPROT database. In this regard our dataset is biased by the available sequence information) mostly share a high sequence similarity among various species, subsequently sharing a high level of structural similarity at the backbone level. Also there is over-representation of some species such as *Streptococcus, Lactobacillus *in our available dataset as shown in Additional file 3: Figure S2. However, among these 202 LuxS proteins we find a wide variety of bacterial species such as the pathogens, commensals, probiotics and the most prominent representatives of the human intestinal microbiota (bacteroidetes and firmicutes). In a nutshell, our analysis reveals the extent of finer structural diversity that can remain hidden within the structure of the same protein from different organisms with the same quaternary association and the same fold at the backbone level. Our method is robust enough to capture minor differences of side-chain orientation at the level of quaternary association, which gives rise to such diversity in function globally. However, LuxS orthologues are present in many more organisms. Metagenomic approaches, in which the genetic material collected from various environments is cloned directly into surrogate hosts, has given us a key to the huge repository of genetic diversity in bacteria. Such approaches will definitely give an even more descriptive idea about the structural conservation[32] of the AI-2 precursor protein in the human gut, bustling with a microbial agora.

Our analysis focuses on the structural properties of a single protein across a dataset to summarize its effect on the typical phenotypic/biological features. However the recent metagenomic data curations have suggested the presence of trillions of organisms in the human intestinal microbiota[33,34]. But not all of these organisms contain the LuxS proteins and AI-2 does not have a signalling role in all the organisms. This definitely narrows down the complexity but then also the system is too complex to be predicted at the structural level of a single protein. A systems biology approach considering the whole network of events has to be employed to decipher the complex puzzle of the human intestinal microbiota. Our method is a contribution to this converging pipeline in terms of the fact that our classification at the structural level can be used to group similar categories for a larger dataset. But nevertheless to understand the overall machinery of LuxS activity and to target LuxS clinically, we need a more holistic view at the systems level.

## Conclusion

The protein structure network graphs are constructed for the 23 LuxS proteins considered in our dataset. Our results indicate presence of completely/partially conserved signature motifs at the dimer interface of the LuxS protein for class (**II–VI**) in relation to their biological function. Our results show the presence of an ensemble of residues at the active site, all of which are required for the proper functioning of LuxS as S-ribosylhomocysteinelyase and thus potential sites of mutation are predicted. We also identify a definite geometric pattern in the form of an isosceles triangle at the dimer interface which we predict to be important for LuxS function. Also, a mini-triad formed by the residues of both the subunits, arising from a stretch of five residue amino acids is found at the apex of the dimer (for class **I–II**, and class **IV–VI**) and is predicted to have structural and thus, functional implications which would call for experimental verifications. This mini-triad on an exposed loop of the protein may be a potential epitope for the design of specific inhibitors of LuxS enzyme. Our characterization of the dimer interface for the probiotic LuxS also makes prediction of their notable characteristic structural features. Also the results of hub analysis reveal the presence of a core hub that is characteristic of the LuxS from extremophiles (class **IV**) and are proposed to contribute robustness to the system. So our analysis emphasizes the importance of the implications of differences in side-chain orientation on functional diversity. To conclude, our study is an attempt to bridge the structure of LuxS protein to its function with special emphasis on the interactions at the dimer interfaces; and hence towards the general phenomenon of quorum sensing in bacteria.

## Methods

### Dataset

In our present study, LuxS proteins from 23 different organisms are considered. Among the LuxS proteins chosen for our study, only 4 structures are known crystallographically. The other structures are generated by homology modelling[35,36] using MODELLER (version 9v3). MODELLER has been used previously to predict structures of pathogenic proteins in relation to protein-protein interactions[37]. Table 1 presents a brief summary of the dataset chosen for our study. With the LuxS protein being widely distributed among the prokaryotes, it was suggested that there might be a role of LuxS in infection, as has also been proposed by the experimental studies that showed LuxS has an effect on pathogenesis. Two potential roles have been proposed for LuxS and its descendent AI-2: Metabolism and Quorum sensing[21,38]. On the basis of the proposed effect of LuxS on pathogenesis and the general biological activity of the organism, our dataset is divided into six distinct classes as summarized in Table 1.

### Modelling and minimization of the LuxS proteins with unknown structures

Among the 23 structures of LuxS proteins chosen for our study, only 4 structures are obtained from the Protein Data Bank. Due to the lack of crystallographically available structures for the rest, they are modelled using MODELLER 9v3 using the standard single-template modelling protocol[35]. The choice of template files is done on the basis of sequence identity of the query proteins with the four proteins from the LuxS family whose structures are known in PDB, as summarized in Table 4. The biologically active form of LuxS is homodimer in each of the above cases and hence the structures are modelled as dimers with a dimeric template to ensure correct interface organization. The modelled LuxS structures are energetically minimized in 1000 steps using AMBER8[39] to ensure that we optimized the structures maximally before undertaking the interface cluster analysis. It is noted that there is a small increase in the backbone level rmsd between the modelled structures on minimisation leading to further increase in side chain rmsd. However, even before minimisation, the interface clusters reflect the differences in side-chain orientation as it is a sensitive tool to judge slight differences in side-chain orientation even if the backbone level rmsd is very close. The LuxS structures are then subjected to subsequent analysis using a graph theoretical approach.

**Table 4:** Summary of templates chosen for the modelling of LuxS structures and the corresponding percentage of sequence identity

Query	**m1vib**	**m1clo**	**m1pyo**	**m1shi**	**m1ste**	**m1stm**	**m1cam**	**m1eco**	**m1sta**	**m1bac**	**m2bac**	**m3bac**	**m1geo**	**m1psy**	**m1the**	**m1lac**	**m2lac**	**m3lac**	**m1bfi**
Template	1j6w	1inn	1inn	1j6w	1stm	1inn	1j6w	1j6w	1j6x	1j98	1j98	1j98	1inn	1j6w	1inn	1inn	1j6w	1inn	1inn

Sequence identity (%)	74.1	50.3	44.0	68.9	44.7	42.0	70.1	68.3	68.5	82.8	69.9	82.2	85.2	68.9	61.0	50.3	52.4	52.4	45.8

### Network construction

Protein structure network graphs are constructed by considering amino acid residues as *nodes *and *edges *are constructed between the nodes on the basis of non-covalent interactions between them (as evaluated from the normalized number of contacts between them) for each LuxS protein [12]. A group of interconnected nodes is defined as a *cluster *and an *interface cluster *is defined as the one with at least one amino acid residue coming from a different protein chain. *Contact number *is defined as the number of edges made by a node; the nodes with contact number equal to four or more are identified as *hubs*. A hub with at least one residue belonging to a different chain in the multimer is referred to as an *interface hub*.

### Evaluation of non-covalent interactions

The non-covalent interaction between side chain atoms of amino acid residues (with the exception of Gly where C_α _atom) are considered, ignoring the interaction between sequence neighbours. The interaction between two residues i and j has been quantified previously in our lab as:

$${\text{I}}_{\text{ij}}=\frac{{\text{n}}_{\text{ij}}}{\sqrt{\left({\text{N}}_{\text{i}}\times {\text{N}}_{\text{j}}\right)}}\times 100$$

where n_ij _is number of distinct atom pairs between the side chains of amino acid residues i and j, which come within a distance of 4.5 Å and N_i _and N_j _are the normalization factors for residues i and j [12].

### Contact criterion on the basis of user-defined interaction strength

The amino acid residues having interaction strength greater than a user-defined cut-off (I_ij _> I_min_) are connected by edges to give a protein structure network (PSN) graph for a given interaction strength I_min_. Such PSNs are constructed at various I_min _values (5%, 6%, and 8%) for all the LuxS structures. A higher I_min _physically indicates a stronger interaction between the residues connected and a lower I_min _indicates a weaker interaction between the connected residues.

### Cluster and Hub Analysis

The protein structure graph is represented as an NXN adjacency matrix, where N is the number of residues in the protein. Each ij^th ^element in this matrix is either 0 or 1 depending on whether the nodes i and j are connected, i.e. interacting or not, on the basis of the chosen I_min_. The amino acids forming disjoint clusters (with a minimum of three residues in each clusters) are identified from the adjacency matrix using a standard graph algorithm (Depth first search (DFS) algorithm)[12]. This gives the clusters of interacting residues in the protein and from this the interface clusters are identified. Similarly, hubs are identified for the residues with contact number greater or equal to four, from which the interface hubs are identified.

The analysis of the structure is then done focussing on the amino acid residue clusters and hubs at I_min _= 6%, in terms of the protein-protein interface clusters. The structurally superposed residues in the interface clusters are identified by aligning (using the program ALIGN[40]) the backbone atoms of the LuxS proteins within a particular class and then plotting the residues in the interface clusters (using VMD)[41]. The above method is repeated for all the classes in our dataset and led to the observation that there are definite signature motifs of sequentially apart but structurally proximal amino acid residues on the interface for each class of LuxS proteins, the classes being defined earlier. The backbone rmsds for each pair within a particular class and across the classes of LuxS protein are evaluated (Additional file 1: Table S2). We also went ahead to investigate the nature of residues in the interface clusters and their positions in the sequence.

## Abbreviations

DPD: 4,5-dihydroxy-2,3-pentanedione; PSN: Protein Structure Network; DFS: Depth First Search; SAM: S-adenosylmethionine; SAH: S-adenosylhomocysteine; SRH: S-ribosylhomocysteine.

## Authors' contributions

Development of the concepts: MB and SV, performed the calculations: MB, analysis of the data and writing the paper: MB and SV.

## Supplementary Material

Additional file 1 Supplementary tables. **Table S1**: Summary of interface cluster analysis of the proteins considered in our dataset at I_min _= 6%. **Table S2**: RMSD values (backbone rmsd) of LuxS protein from 23 organisms in our dataset. **Table S3**: Detailed description of residues present in the interface clusters at I_min _= 6% for Class (I–VI). **Table S4**: Description of the hubs present in LuxS from extremophiles at different I_min _values (the numbering of residues are done according to sequence). **Table S5**: Various bacterial species and the mini-triad motif associated with them across our dataset. **Table S1**: The total number of interface clusters vary from 4–12 and the total number of residues in the interface clusters vary from 36 to 73. The mini-triad pattern for each protein is also given in the table with the physiological manifestations of LuxS mutation in each class (except III, IV and V) except for *T. thermophilus *(class IV) in which the mini-triad pattern is absent from the sequence. **Table S2**: It can be readily noted that all the rmsd values are < 1.5, indicating the similarity in the backbone structures for the proteins in our dataset. Bold highlights indicate the rmsd values within members of a particular class. **Table S3**: Some clusters have been merged together for simplicity of representation. **Table S4**: The table reveals the presence of hubs at higher I_min _values for class IV; for m1psy (psychrophile), 1inn and m1geo (extreme radiation-resistent) and m1the (hyperthermophile). They all belong to the broad category of extremophiles. **Table S5**: Various bacterial species and the mini-triad motif associated with them across our dataset. The absolute mini-triad motifs are indicated without 'X'. Here GKD essentially means GXKXD. Click here for file

Additional file 2 **Figure S1(a-b)**. **Figure S1**: All interface amino acid clusters for the members of (a) class II and (b) class III are plotted on the superposed backbones of the four LuxS structures within class II and III respectively. The residues are represented as van der Waal's spheres and the backbones are given in transparent new cartoon. The figures show an overall uniform topological orientation of all the interface clusters for members within a class; the class III interface clusters lack the mini-triad and the connecting bridge between the two subunits in the lower half of the protein in contrast to that shown in class II. So they share a distinct overall distribution as becomes evident from the figure. Click here for file

Additional file 3 **Figure S2**: Frequency of occurrence of various bacterial species in our dataset consisting of 202 LuxS proteins. The abbreviations used for bacterial species are a part of the UNIPROT id; e.g. LUXS_BACSU means LuxS from *Bacillus *(species) *subtilis *(organism). We have indicated the species by three letter codes e.g. BAC in Figure S2. Click here for file
