# Supplementary material for: Functional correlation of bacterial LuxS with their quaternary associations: interface analysis of the structure networks
Source: BMC Struct Biol. 2009 Feb 25;9:8. doi: 10.1186/1472-6807-9-8 (PMC2656534; doi:10.1186/1472-6807-9-8)
Supplement: Additional file 1 — Supplementary tables. Table S1: Summary of interface cluster analysis of the proteins considered in our dataset at Imin = 6%. Table S2: RMSD values (backbone rmsd) of LuxS protein from 23 organisms in our dataset. Table S3: Detailed description of residues present in the interface clusters at Imin = 6% for Class (I–VI). Table S4: Description of the hubs present in LuxS from extremophiles at different Imin values (the numbering of residues are done according to sequence). Table S5: Various bacterial species and the mini-triad motif associated with them across our dataset. Table S1: The total number of interface clusters vary from 4–12 and the total number of residues in the interface clusters vary from 36 to 73. The mini-triad pattern for each protein is also given in the table with the physiological manifestations of LuxS mutation in each class (except III, IV and V) except for T. thermophilus (class IV) in which the mini-triad pattern is absent from the sequence. Table S2: It can be readily noted that all the rmsd values are < 1.5, indicating the similarity in the backbone structures for the proteins in our dataset. Bold highlights indicate the rmsd values within members of a particular class. Table S3: Some clusters have been merged together for simplicity of representation. Table S4: The table reveals the presence of hubs at higher Imin values for class IV; for m1psy (psychrophile), 1inn and m1geo (extreme radiation-resistent) and m1the (hyperthermophile). They all belong to the broad category of extremophiles. Table S5: Various bacterial species and the mini-triad motif associated with them across our dataset. The absolute mini-triad motifs are indicated without 'X'. Here GKD essentially means GXKXD. [file 1472-6807-9-8-S1.pdf]

SUPPLEMENTARY TABLES

Table S1: Summary of interface cluster analysis of the proteins considered in our dataset at I_{min} = 6%

CLASS	NAME OF ORGANISM	TOTX(total no. Of interface clusters)	TOTRES(total no. Of residues in interface clusters)	Mini-triad motif	CLASSIFICATION
I	1j6w	5	36	TXKXD	LuxS mutation affects biofilm development
	1ste	4	56	GXNXD	
	1stm	8	60	GXKXD	
II	1j6x	6	69	GXNXD	LuxS mutation affects metabolism/motility etc.
	1eco	8	55	TXHXD	
	1cam	7	45	TXKXD	
	1sta	6	73	GXNXD	
III	1j98	7	38	VXTXG	LuxS *fromBacillus sp.*
	1bac	7	45	VXSXG	
	2bac	10	56	VXSXG	
	3bac	9	67	VXTXG	
IV	1inn	7	45	TXKXD	LuxS from extremophiles
	1geo	6	33	TXRXD	
	1psy	8	50	TXSXD	
	1the	9	55	--------	
V	1vib	12	56	TXKXD	LuxS mutation affects toxin production
	1clo	8	52	GXKXD	
	1shi	7	52	TXHXD	
	1pyo	10	58	GXKXD	
VI	1lac	9	65	GXKXD	LuxS from Probiotics
	2lac	7	50	GXKXD	
	3lac	6	38	GXKXD	
	1bfi	10	61	GXHXD	

The total number of interface clusters vary from 4-12 and the total number of residues in the interface clusters vary from 36 to 73. The mini-triad pattern for each protein is also given in the table with the physiological manifestations of LuxS mutation in each class (except III, IV and V) except for T.thermophilus (class IV) in the mini-triad pattern is absent from the sequence.

Table S2: Backbone RMSD values of 23 pairs of LuxS proteins from different organisms (from class I-VI)

**rmsd**	**1vib**	**1clo**	**1shi**	**1pyo**	**1j6w**	**1ste**	**1stm**	**1j6x**	**1eco**	**1cam**	**1sta**	**1lac**	**2lac**	**3lac**	**1bfi**	**1inn**	**1geo**	**1psy**	**1the**	**1j98**	**1bac**	**2bac**	**3bac**
**1vib**	**0**	**1.04**	**0.42**	**1.10**	0.60	1.28	1.11	1.40	0.40	0.42	1.32	1.06	1.07	1.04	1.10	1.14	1.09	0.41	1.06	1.18	1.22	1.15	1.20
**1clo**	**1.04**	**0**	**1.00**	**0.49**	1.14	1.09	0.56	1.21	1.01	1.01	1.10	0.53	0.51	0.48	0.50	0.62	0.43	1.05	0.48	1.08	1.10	1.06	1.17
**1shi**	**0.42**	**1.00**	**0**	**1.06**	0.55	1.24	1.08	1.36	0.33	0.40	1.32	1.03	1.04	1.02	1.06	1.09	1.05	0.38	1.03	1.18	1.23	1.16	1.24
**1pyo**	**1.10**	**0.49**	**1.06**	**0**	1.26	1.21	0.48	1.31	1.06	1.06	1.21	0.54	0.49	0.49	0.50	0.72	0.51	1.10	0.51	1.19	1.19	1.19	1.21
**1j6w**	0.60	1.14	0.55	1.26	**0**	**1.35**	**1.24**	1.42	0.56	0.62	1.42	1.23	1.19	1.22	1.23	1.20	1.18	0.55	1.20	1.20	1.28	1.23	1.26

**1ste**	1.28	1.o9	1.24	1.21	**1.35**	**0**	**1.27**	0.45	1.23	1.23	0.39	1.20	1.20	1.18	1.13	1.18	1.23	1.21	1.18	0.73	0.76	0.75	0.77
**1stm**	1.11	0.56	1.08	0.48	**1.24**	**1.27**	**0**	1.37	1.10	1.12	1.26	0.50	0.50	0.52	0.56	0.75	0.53	1.13	0.53	1.28	1.32	1.24	1.31
**1j6x**	1.40	1.21	1.36	1.31	1.42	0.45	1.37	**0**	**1.35**	**1.34**	**0.43**	1.35	1.30	1.34	1.27	1.26	1.34	1.35	1.32	0.78	0.84	0.83	0.88
**1eco**	0.40	1.01	0.33	1.06	0.56	1.23	1.10	**1.35**	**0**	**0.38**	**1.30**	1.04	1.02	1.02	1.05	1.10	1.07	0.38	1.04	1.17	1.22	1.16	1.19
**1cam**	0.42	1.01	0.40	1.06	0.62	1.23	1.12	**1.34**	**0.38**	**0**	**1.28**	1.07	1.08	1.08	1.09	1.14	1.09	0.38	1.09	1.13	1.22	1.13	1.17
**1sta**	1.32	0.53	1.32	1.21	1.42	0.39	1.26	**0.43**	**1.30**	**1.28**	**0**	1.22	1.22	1.23	1.14	1.22	1.22	1.28	1.21	0.72	0.77	0.76	0.79
**1lac**	1.06	0.53	1.03	0.54	1.23	1.20	0.50	1.35	1.04	1.07	1.22	**0**	**0.52**	**0.51**	**0.52**	0.71	0.52	1.08	0.54	1.21	1.24	1.17	1.24
**2lac**	1.07	0.51	1.04	0.49	1.19	1.20	0.50	1.30	1.02	1.08	1.22	**0.51**	**0**	**0.42**	**0.50**	0.66	0.50	1.05	0.53	1.23	1.25	1.21	1.23
**3lac**	1.04	0.48	1.02	0.49	1.22	1.18	0.52	1.34	1.02	1.08	1.23	**0.52**	**0.42**	**0**	**0.44**	0.59	0.48	1.06	0.52	1.26	1.23	1.21	1.22
**1bfi**	1.10	0.50	1.06	0.50	1.23	1.13	0.56	1.27	1.05	1.09	1.14	**0.51**	**0.50**	**0.44**	**0**	0.64	0.52	1.10	0.53	1.15	1.22	1.16	1.20
**1inn**	1.14	0.62	1.09	0.72	1.20	1.18	0.75	1.26	1.10	1.14	1.22	0.71	0.66	0.59	0.64	**0**	**0.49**	**1.12**	**0.67**	1.16	1.21	1.16	1.26
**1geo**	1.09	0.43	1.05	0.51	1.18	1.23	0.53	1.34	1.07	1.09	1.22	0.52	0.50	0.48	0.52	**0.49**	**0**	**1.10**	**0.44**	1.17	1.17	1.17	1.22
**1psy**	0.41	1.05	0.38	1.10	0.55	1.21	1.13	1.35	0.38	0.38	1.28	1.08	1.05	1.06	1.10	**1.12**	**1.10**	**0**	**1.08**	1.18	1.19	1.11	1.18
**1the**	1.06	0.48	1.03	0.51	1.20	1.18	0.53	1.32	1.04	1.09	1.21	0.54	0.53	0.52	0.53	**0.67**	**0.44**	**1.08**	**0**	1.18	1.19	1.16	1.22
**1j98**	1.18	1.08	1.18	1.19	1.20	0.73	1.28	0.78	1.17	1.13	0.72	1.21	1.23	1.26	1.15	1.16	1.17	1.18	1.18	**0**	**0.40**	**0.44**	**0.37**
**1bac**	1.22	1.10	1.23	1.19	1.28	0.76	1.32	0.84	1.22	1.22	0.77	1.24	1.25	1.23	1.22	1.21	1.17	1.19	1.19	**0.40**	**0**	**0.46**	**0.35**
**2bac**	1.15	1.06	1.16	1.19	1.23	0.75	1.24	0.83	1.16	1.13	0.76	1.17	1.21	1.21	1.16	1.16	1.17	1.11	1.16	**0.44**	**0.46**	**0**	**0.45**

**3bac**	1.20	1.17	1.24	1.21	1.26	0.77	1.31	0.88	1.19	1.17	0.79	1.24	1.23	1.22	1.20	1.26	1.22	1.18	1.22	**0.37**	**0.35**	**0.45**	**0**

It can be readily noted that all the values are < 1.5, indicating the similarity in the backbone structures for the proteins in our dataset. Bold highlights indicate the rmsd values within members of a particular class.

Table S3: Detailed description of residues present in the interface clusters at Imin = 6% for Class (I-VI)

CLUSTER NO.	1VIB		1J6W		1J6X		1LAC		1J98		1INN	
	CHAIN A	CHAIN B	CHAIN A	CHAIN B	CHAIN A	CHAIN B	CHAIN A	CHAIN B	CHAIN A	CHAIN B	CHAIN A	CHAIN B
ACTIVE SITE I	82G, 7F, 4L	54H, 57E, 134H, 123L, 120I, 55T, 58H, 122E	5F, 2L, 77C	50H, 54H, 126H, 53E, 115L, 112I, 51T, 114E	3V, 6F, 2N, 46H, 76Q	51H, 55H, 122H, 59E, 121N, 118W, 52S, 108V, 56L, 110A	4V, 7F, 5E, 79C, 23T	54H, 58H, 130H, 55T, 65R, 71Y, 64L, 103S, 85L, 36F, 38L	1V, 4F	51H, 55H, 128H, 52T, 114I, 130L, 134K, 131E, 138R, 116A	5F, 2V	56H, 52H, 55E, 121H, 53T, 107I, 110V, 109G
ACTIVE SITE II	54H, 57E, 134H, 123L, 58H, 122E	82G, 7F, 4L	50H, 54H, 126H, 53E, 115L, 112I, 51T, 114E	5F, 2L, 77C	51H, 55H, 122H, 59E, 121N, 118W, 62R, 68V, 61I, 110A, 116C	46H, 76Q, 6F, 2N, 3V	------	------	51H, 54E, 55H, 116A, 128H, 52T, 114I	1V, 4F, 80G	---------	---------
APEX CLUSTER	27T, 29K, 31D	27T, 29K, 31D	23T, 25K, 27D	23T, 25K, 27D	26G, 28N, 30D, 85N, 84L,	27V, 28N, 30D, 85N	28Q, 29K, 31D	28Q, 29K, 31D	------	------	25T, 27K, 29D	25T, 27K, 29D
IV	10D, 13R, 161L, 12T, 126Y	126Y, 125E, 10D, 13R, 161L	43I, 78R, 125E, 47K	43I, 78R, 125E, 47K	4E, 19R, 36D, 17Y, 38R, 77T, 144V, 8L, 80Y, 34K, 22D, 82T, 69V, 70D	70D, 24K, 4E, 19R, 36D, 17Y, 38R, 77T, 144V, 8L, 80Y, 34K	10D, 13K, 158E	122E	24V	24V, 74I, 89S	8D, 11K, 144I, 146L	113L

V	20R, 9V, 37D, 81M, 88Y, 156N	77D, 65R, 75I, 89M			9D, 12K	114K	14V, 18Y, 153E, 15K, 37D, 20R, 9L, 155H, 84H, 35N, 22I, 77F, 39R, 81T	57E	81Q, 124Q, 127L	81Q, 124Q, 127L	70D, 33K, 79Y	3E, 23K, 33K, 79Y, 70D
VI	47I, 84R	133M, 130T,					33I, 86L, 88W, 72I, 25E	33I, 88W	119E, 126K	41N	76R	120D, 117N
VII	74E, 92I	25M					50T, 129D, 126N, 80R	80R, 50T, 129D, 126N	75D	22H, 32K	112E, 118Y	42N
VIII	50E	47I, 84R					75S	75S, 84H, 35N, 23T, 7F, 86L, 72I, 5E				

Some clusters have been merged together for simplicity of representation.

Table S4: Description of the hubs present in LuxS from extremophiles at different Imin values (the numbering of residues are done according to sequence)

Protein code	Imin	Total no. Of Hubs	Centre of Hub	Hub Residues
1inn	6%	4	21Y(A)	{17V, 151Q, 40D, 42R} (A)
			131H(A)	{57H, 61H, 58T, 117I,120V} (A)
			21Y(B)	{17V, 151Q, 40D, 42R} (B)
			131H(B)	{57H, 61H, 58T, 117I,120V} (B)
	8%	3	21Y(A)	{17V, 151Q, 40D, 42R} (A)

			131H(A)	{57H, 61H, 58T, 117I,120V} (A)
			21Y(B)	{17V, 151Q, 40D, 42R} (B)
	10%	2	21Y(A)	{17V, 151Q, 40D, 42R} (A)
			21Y(B)	{17V, 151Q, 40D, 42R} (B)
	12%	1	21Y(A)	{17V, 151Q, 40D, 42R} (A)
1geo	6%	3	18Y (A)	{14V, 148Q, 37D, 39R} (A)
			39R (A)	{18Y, 11H, 77M, 81T} (A)
			18Y (B)	{14V, 148Q, 37D, 39R} (B)
	8%	2	18Y (A)	{14V, 148Q, 37D, 39R} (A)
			18Y (B)	{14V, 148Q, 37D, 39R} (B)
	10%	1	18Y (B)	{14V, 148Q, 37D, 39R} (B)
	12%	1	18Y (B)	{14V, 148Q, 37D, 39R} (B)
1psy	6%	5	39R (A)	{11H,14M, 81M, 85T} (A)
			60Y (A)	{38L, 104W, 40F, 64M} (A)
			104W (A)	{38L, 60Y, 40F, 108M} (A)
			134H (A)	{58H, 123L, 54H, 55T} (A)
			39R (B)	{11H,14M, 81M, 85T} (B)

	8%	1	134H (A)	{58H, 123L, 54H, 55T} (A)
1the	6%	2	18Y (A)	{14V, 148Q, 37D, 39R} (A)
			18Y (B)	{14V, 148Q, 37D, 39R} (B)
	8%	2	18Y (A)	{14V, 148Q, 37D, 39R} (A)
			18Y (B)	{14V, 148Q, 37D, 39R} (B)
	10%	2	18Y (A)	{14V, 148Q, 37D, 39R} (A)
			18Y (B)	{14V, 148Q, 37D, 39R} (B)
	11%	1	18Y (B)	{14V, 148Q, 37D, 39R} (B)

The table reveals the presence of hubs at higher Imin values for this class for 1psy (psychrophile), 1inn and 1geo (extreme radiation-resistent) and 1the (hyperthermophile). They all belong to the broad category of extremophiles.

Table S5

**MINI-TRIAD MOTIF**	**BACTERIAL SPECIES (frequency of contribution)**
GKD	STR(22) LAC(15) CLO(4)
TKD	SHE(16) CAM(9) HAE(7) VIB(6) NEI(5) ACT(2) HEL, PAS, ARC, DEI, PHO, MAN, MAR, WOL(1)
TND	AER(2)
VTG	BAC(4)
VSG	BAC(7) ARC, GEO(1)
ISG	BAC(1) GEO(1)
EGE	BAC(1)
GHD	LIS(4) BIF(2) CHR, DIC(1)
TEV	BOR(3)

THD	ECO(9) YER(7) SAL(5) SHI(5) SER(2) CIT, PHO, ENT, ERW, SOD(1)
KAN	DES(1)
KGV	CLO(1)
TRD	DEI(1)
NDA	ENT(1)
TSD	ENT, PRO, PSY, SHE, SUL(1)
GND	STA(18) HEL(4) OCE(1)
GNG	LEU(1)
GKG	OEN(1)
YGE	POR(1)
LAD	PRO(1)
GVD	STR(3)
TCD	SUL(1)
LG-	THE(2)
TGD	THI(1)

Various bacterial species and the mini-triad motif associated with them across our dataset. The number of species with a particular motif is given in the parenthesis.
