# Supplementary figures and images for: Functional correlation of bacterial LuxS with their quaternary associations: interface analysis of the structure networks
Source: BMC Struct Biol. 2009 Feb 25;9:8. doi: 10.1186/1472-6807-9-8 (PMC2656534; doi:10.1186/1472-6807-9-8)

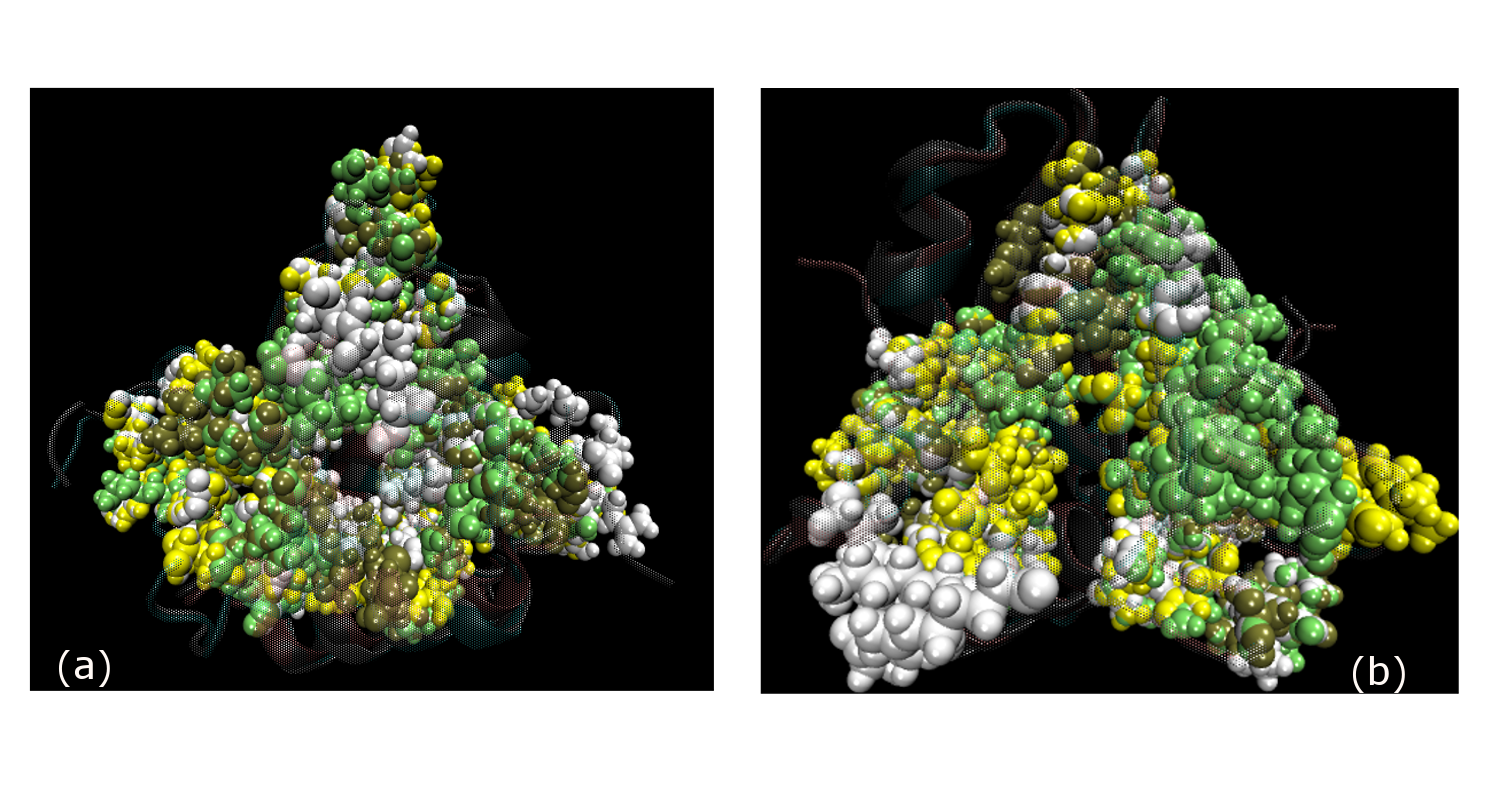

Supplement: Additional file 2 — Figure S1(a-b). Figure S1: All interface amino acid clusters for the members of (a) class II and (b) class III are plotted on the superposed backbones of the four LuxS structures within class II and III respectively. The residues are represented as van der Waal's spheres and the backbones are given in transparent new cartoon. The figures show an overall uniform topological orientation of all the interface clusters for members within a class; the class III interface clusters lack the mini-triad and the connecting bridge between the two subunits in the lower half of the protein in contrast to that shown in class II. So they share a distinct overall distribution as becomes evident from the figure. [file 1472-6807-9-8-S2.tiff]

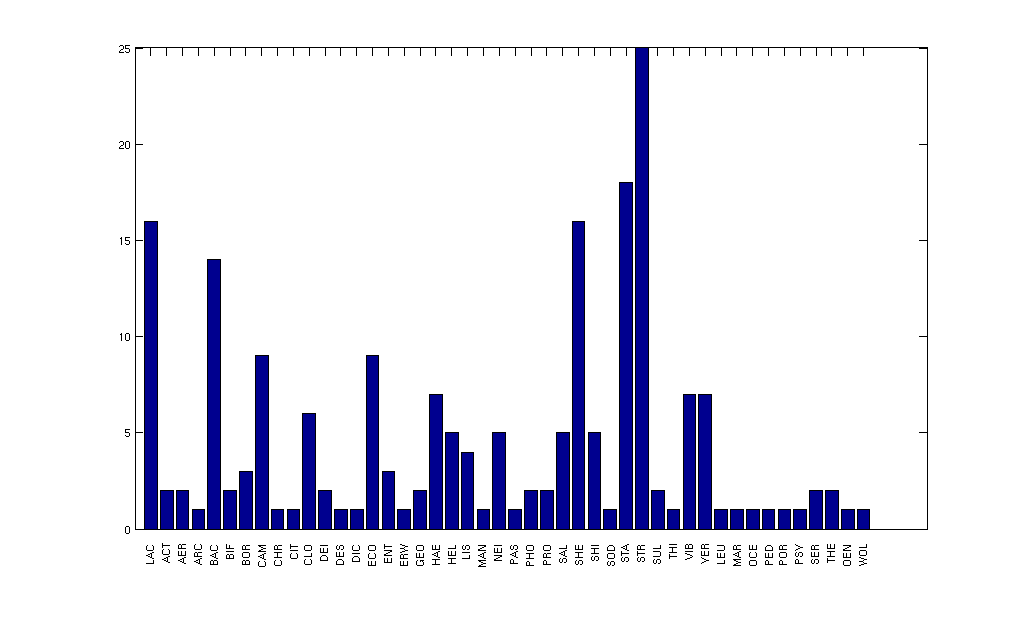

Supplement: Additional file 3 — Figure S2: Frequency of occurrence of various bacterial species in our dataset consisting of 202 LuxS proteins. The abbreviations used for bacterial species are a part of the UNIPROT id; e.g. LUXS_BACSU means LuxS from Bacillus (species) subtilis (organism). We have indicated the species by three letter codes e.g. BAC in Figure S2. [file 1472-6807-9-8-S3.tiff]
